# Identifying Cardiac Syncope Based on Clinical History: A Literature-Based Model Tested in Four Independent Datasets

**DOI:** 10.1371/journal.pone.0075255

**Published:** 2013-09-24

**Authors:** Janneke Berecki-Gisolf, Aaron Sheldon, Wouter Wieling, Nynke van Dijk, Giorgio Costantino, Raffaello Furlan, Win-Kuang Shen, Robert Sheldon

**Affiliations:** 1 Libin Cardiovascular Institute of Alberta, University of Calgary, Calgary, Alberta, Canada; 2 Faculty of Veterinary Medicine, University of Calgary, Calgary, Alberta, Canada; 3 Departments of Internal Medicine, Academic Medical Center, University of Amsterdam, Amsterdam, The Netherlands; 4 General Practice/Family Medicine, Academic Medical Center, University of Amsterdam, Amsterdam, The Netherlands; 5 Syncope Unit, Internal Medicine II, “L. Sacco” Hospital, University of Milan, Milan, Italy; 6 Division of Cardiovascular Diseases, Mayo Clinic, Rochester, Minnesota, United States of America; University Heart Center Freiburg, Germany

## Abstract

**Background:**

We aimed to develop and test a literature-based model for symptoms that associate with cardiac causes of syncope.

**Methods and Results:**

Seven studies (the derivation sample) reporting ≥2 predictors of cardiac syncope were identified (4 Italian, 1 Swiss, 1 Canadian, and 1 from the United States). From these, 10 criteria were identified as diagnostic predictors. The conditional probability of each predictor was calculated by summation of the reported frequencies. A model of conditional probabilities and a priori probabilities of cardiac syncope was constructed. The model was tested in four datasets of patients with syncope (the test sample) from Calgary (n=670; 21% had cardiac syncope), Amsterdam (n=503; 9%), Milan (n=689; 5%) and Rochester (3877; 11%). In the derivation sample ten variables were significantly associated with cardiac syncope: age, gender, structural heart disease, low number of spells, brief or absent prodrome, supine syncope, effort syncope, and absence of nausea, diaphoresis and blurred vision. Fitting the test datasets to the full model gave C-statistics of 0.87 (Calgary), 0.84 (Amsterdam), 0.72 (Milan) and 0.71 (Rochester). Model sensitivity and specificity were 92% and 68% for Calgary, 86% and 67% for Amsterdam, 76% and 59% for Milan, and 73% and 52% for Rochester. A model with 5 variables (age, gender, structural heart disease, low number of spells, and lack of prodromal symptoms) was as accurate as the total set.

**Conclusion:**

A simple literature-based Bayesian model of historical criteria can distinguish patients with cardiac syncope from other patients with syncope with moderate accuracy.

## Introduction

The differential diagnosis of syncope is wide, ranging from vasovagal syncope, to cardiac arrhythmias, orthostatic hypotension, and valvular heart disease [1]. Among patients less than 65 years of age, cardiac syncope comprises little more than one-fifth of causes of syncope, whereas in patients above 65 years it is the cause of syncope in 42% of cases [2,3]. Importantly, patients with cardiac syncope are at increased risk of cardiovascular events and mortality [4].

Several studies have investigated the usefulness of clinical history and demographics as predictors of the cause of syncope [5-8]; others have investigated criteria associated with adverse outcome in syncope patients [9-14]. However, these studies differ in setting, outcome, definitions, and potential markers in relation to syncope. The purpose of this study was to use these publications to develop an international, literature-based model to predict cardiac syncope. A model for predicting cardiac syncope based on historical and demographic criteria that is effective and robust to minor differences in definition and setting may provide a useful tool for risk stratification of patients presenting with syncope.

Accordingly, we conducted a literature search to identify studies reporting historical and demographic variables associated with cardiac syncope, and combined the reported results in a conditional probabilities model. We then tested the effectiveness of this model against data of patients with syncope from four centres that were not associated with the derivation populations.

## Methods

Model derivation and model testing were conducted independently. Two samples of publications were defined. Publications were included in the *derivation sample* [5,6,15-19] if they presented summary findings such as proportions or sensitivity and specificity, but the patient-specific primary data sets were not acquired by the investigators. Publications were included in the *test sample* if they had summary data, and the patient-specific primary data sets were acquired by the investigators [7-9,20,21].

Our intent was to summarize the results from major articles published in the field. Pubmed was searched for relevant studies in 2 separate searches. First, the terms “diagnosis”, “signs and symptoms” and “vasovagal syncope” were entered. Second, the terms “clinical history”, “diagnosis” and “syncope” were entered. Resulting abstracts were scanned manually and studies were included if they featured all of the following: patients with ≥1 transient loss of consciousness; a diagnosis of cardiac syncope vs. other causes, according to the degree of evidence accepted in each paper; and ≥2 historical symptoms or criteria (not counting results of physical exam or further diagnostics) reported in relation to the final diagnosis. Secondary searches were conducted in local bibliographies and in references from identified articles.

### Key variables in the derivation sample

Variables from the 7 derivation populations [5,6,15-19] were selected as predictors if they were reported to be associated with specific causes of syncope. Similarities and differences among the derivation datasets in the prevalence of predictors, and the relation between predictors and outcome, were identified using logistic regression models. Some studies included a range of variables in a multiple logistic regression model without presenting a cross-tabulation of the presence of individual variables against the final diagnosis; therefore a statistically significant association between a predictor and the diagnosis may have been reported without a quantification of this association. To ensure data were entered in the model on all selected predictors, we introduced an arbitrary threshold: only variables reported to be statistically significantly associated with the diagnosis (regardless of how the results are presented), in all patients or among a subgroup of patients, in ≥3 studies, were included. Signs observed by bystanders were excluded because they are conditional upon the presence of bystanders [22].

### Model construction in derivation sample

The most important model variables were combined in a conditional probabilities model. The conditional probabilities of predictors, given the diagnosis, were derived by summation of the frequencies reported in each study, whether the association was reported as statistically significant or not. Associations that were quantified indirectly (for example from sensitivity and specificity, or mean and standard deviation) were recalculated as frequencies, where possible.

**Figure 1:**
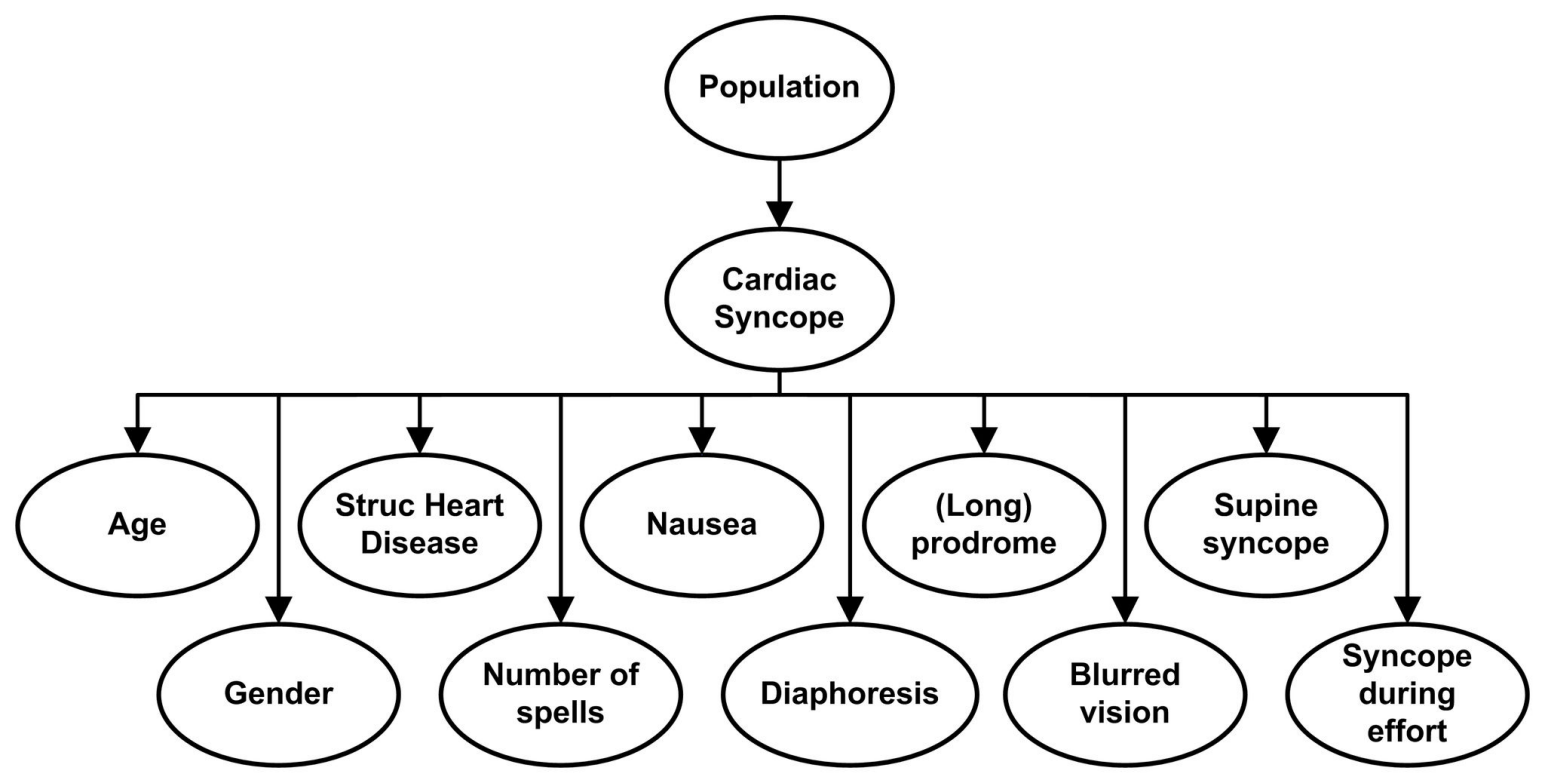
Schematic representation of the joint probability model structure.

The model consists of a graphical structure of nodes, illustrated in Figure 1. The nodes represent the study population, cardiac syncope and a range of predictors. The *a priori* probability of cardiac syncope corresponded with the prevalence of cardiac syncope in the study population. The direction of the arrows indicates that the outcome (cardiac syncope) is dependent on the predictors (age, gender, structural heart disease, etc.) For each symptom, the relation between the symptom and the outcome is characterised by a conditional probability table: the probability that cardiac syncope causes the symptom. For example, based on the literature search we know that the probability of nausea, given the presence of cardiac syncope, is 8%; the probability of nausea, given the presence of non-cardiac syncope, is 20%. The probabilistic information and the node structure are combined to calculate the probability that a patient suffers from cardiac syncope, using a joint Bernouilli probability model. The probability that a patient presenting with a specific set of historical criteria had cardiac syncope is the normalized product of the individual conditional probabilities, multiplied by the prior probability that a random member of the patient population has cardiac syncope. For each patient, the model output is within the range of probability of zero and one.

### Model description

The accuracy of the model in predicting cardiac syncope was tested in datasets from 4 independent syncope populations for whom primary data were available [7-9,20,21]. A graphical representation of the final model structure is given in Figure 1. The population node contains 4 categories; one for each test dataset (Calgary [7,8,20], Amsterdam [21], Milan [9] and Rochester [2]). Each test dataset corresponds to a data-derived *a priori* chance of cardiac syncope. For each test subject, the prior probability of cardiac syncope, which is specific to the test population (e.g. 21% in the Calgary data), is then updated with a posterior probability: the probability of cardiac syncope given the population as well as the predictors for that subject.

### Testing the Model from the Derivation Sample

For each test dataset, patient data were fitted to the conditional probabilities model, resulting in a predicted probability of cardiac syncope for each patient. The predicted probabilities against the actual diagnoses were displayed in a receiver operating characteristics (ROC) curve. A c-statistic was computed to give an overall estimate of how well the model discriminated cardiac vs. non-cardiac syncope in the test data.

### Parsimonious model

Not all test datasets contained all model variables; therefore, for a fair comparison of the model efficiency in the 4 test datasets, model fits were repeated using only the 5 predictors available in all datasets.

### Testing the Model using Resampled Data

Among patients who have only fainted once, and among older patients, historical criteria might be less predictive [17]. Accrual bias might, therefore, have affected the associations between predictors and outcome. We standardized the proportion of older patients, and of patients who only fainted once to attenuate differences due to accrual bias. Each dataset was resampled to create 1000 samples with a standardized distribution of age and number of spells (both as categorical variables), using PROC SURVEYSELECT in SAS. The overall distribution of age and number of spells reported in the literature used to build the model was used to create the standardised distribution. Only variables common to all datasets were used in the resampled data. Hugin 7.1 was used to compile the conditional probabilities model, to establish proof of concept. For computing large datasets, the model was programmed in Matlab using Kevin Murphy’s Bayes Net Toolbox (BNT). All other analyses were conducted using SAS software, version 9.2.

## Results

### Derivation populations

The literature search identified seven studies [5,6,15-19] that reported symptoms in relation to cardiac versus non-cardiac diagnoses (Table 1). Of the 2388 patients 1317 (55%) were from Italy, 939 (39%) from Switzerland, 80 (3%) from the US and 52 (2%) from Canada. Only the patients reported by Galizia et al. [17] were selected based on age; they were at least 65 years old.

**Table 1:** The seven studies on which the conditional probabilities model was based.

Reference	Alboni et al. [5]	Calkins et al. [15]	Del Rosso et al. [16]	Del Rosso et al. [6]	Galizia et al. [17]	Iglesias et al. [18]	Sud et al. [19]
Patient selection	≥1 LOC	≥1 LOC	≥1 LOC	≥1 LOC	≥1 LOC	≥1 LOC or presyncope	≥1 LOC with injury or recurrent LOC
Location	Italy	US	Italy	Italy	Italy	Switzerland	Canada
Clinical setting	Syncope unit	Patients referred for evaluation of syncope	Syncope unit	Emergency department	Admitted or emergency department	Ambulatory syncope clinic	Patients undergoing monitoring for syncope
Patient exclusion		Sick sinus syndrome		Non-syncopal cause of LOC	Severe cognitive impairment, active malignancies, limitation in activities of daily living	Seizure, vertigo or coma	Ejection fraction <35%
Minimum age for inclusion	≥18	≥18		≥18	≥65		
Age (mean±SD)	61±20	58±17	<65: 43±16; ≥65: 76±7	63±21	79±7	52±21	67±14
Number of patients	341	80	485	260	231	939	52
Main diagnostic categories							
Cardiac syncope	CS	AV block; VT	CS	CS	CS	Arrhythmia	Primary arrhythmic
	n=78	n=16, n=32	n=116	n=44	n=34	n=148	n=20
(Predominantly) neurally mediated syncope	Neurally mediated syncope	Vasodepressor	VVS	Non-CS	Non-CS	VV/Psy	Nonarrhythmia
	n=199	n=32	n=296	n=216	n=174	n=387	n=32
Other	Unexplained/ Neurologic/ Psychiatric		Unknown		Unknown	Hypotension Miscellaneous Unexplained	
	n=64		n=73		n=23	n=58, n=27, n=319	

VVS= vasovagal syncope, neurally mediated syncope, neurocardiogenic syncope, CS= cardiac syncope, AV = atrioventricular, VT= ventricular tachycardia. LOC= loss of consciousness.

### Predictors of cardiac syncope

**Figure 2:**
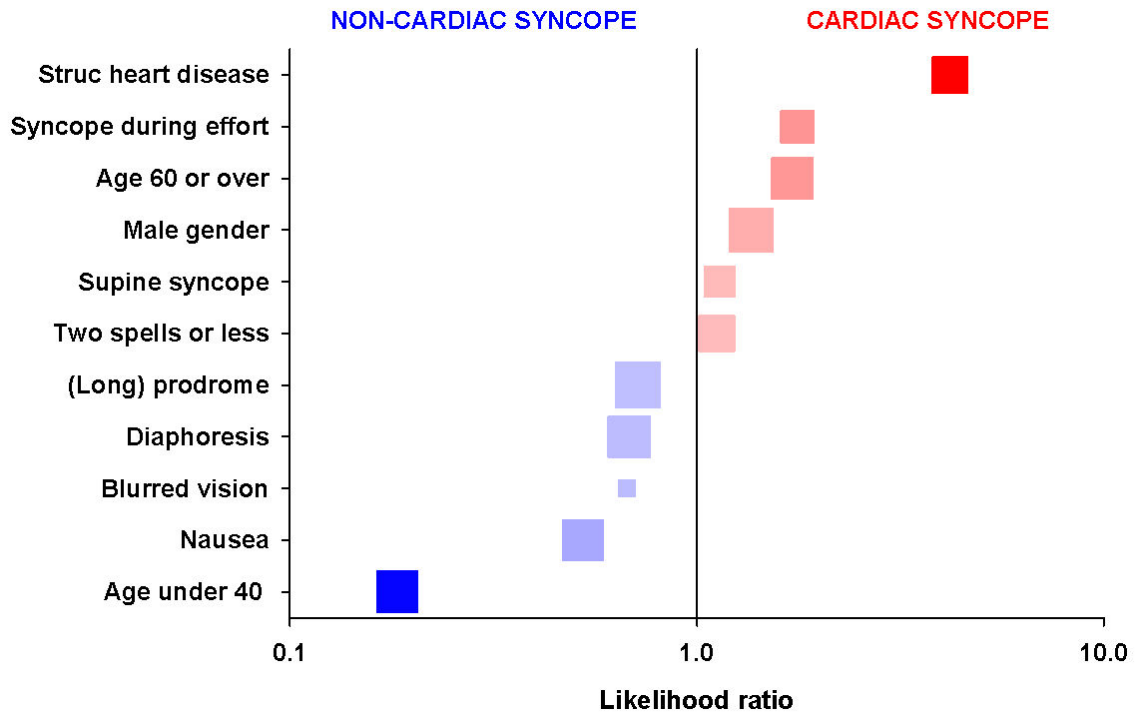
Predictors of cardiac syncope in the derivation populations.

Eleven variables were statistically significantly associated with the diagnosis in 3 or more derivation studies listed in Table 1. The resulting conditional probability tables for these variables are given in Table 2. Palpitations were more common among cardiac syncope patients in some studies [5,6], and more common among vasovagal patients in others [15,18]. Overall the association of palpitations with cardiac vs. non-cardiac syncope was not statistically significant; therefore, this variable was excluded. The resulting 10 variables associated with cardiac syncope were age >60 years, male sex, structural heart disease, <3 spells, and supine syncope and effort syncope. Factors associated with non-cardiac syncope included age < 40 years, a long prodrome, nausea, diaphoresis, and blurred vision. These had moderate accuracy in identifying patients with cardiac syncope (Figure 2).

**Table 2:** Overview of selected published studies: prevalence of selected symptoms in relation to final diagnosis.

	Cardiac syncope								Non-cardiac syncope											
Reference	[5]	[15]	[16]	[6]	[17]	[18]	[19]	Total	[5]	[15]	[16]	[6]	[17]	[18]	[19]	Total	χ^2^ Test			Likeli-hood ratio
Number of patients	78	48	116	44	34	148	20	488 (21%)	259	32	296	216	174	791	32	1800 (79%)	χ^2^	DF	p	
Age																				
<40 years	1*	0*	-	-	-	1*	0*	3 (1%)	50*	13*	-	-	-	259*	1*	234 (29%)	171.7	2	<0.0001	-
40 to 60 years	13	10	-	-	-	37	5	65 (22%)	89	13	-	-	-	315	10	427 (38%)				
≥60 years	64	38	-	-	-	110	15	226 (77%)	120	5	-	-	-	217	21	363 (33%)				
Gender																				
Male	49	41	-	34	12	90	12	238 (64%)	133	10	-	122	74	388	16	743 (49%)	25.4	1	<0.0001	1.30
Female	29	7	-	10	22	58	8	134 (36%)	126	22	-	94	100	403	16	761 (51%)				
Struc heart disease																				
Yes	-	-	107	-	-	-	11	118 (87%)	-	-	77	-	-	-	8	85 (26%)	283.7	1	<0.0001	3.00
No	-	-	9	-	-	-	9	18 (13%)	-	-	219	-	-	-	24	243 (74%)				
Number of spells																				
Two spells or less	-	37	77	-	-	-	-	114 (70%)	-	4	120	-	-	-	-	124 (38%)	44.0	1	<0.0001	1.84
More than two spells	-	11	39	-	-	-	-	50 (30%)	-	28	176	-	-	-	-	204 (62%)				
Nausea																				
Yes	4	2	7	-	2	19	-	34 (8%)	34	16	45	-	38	174	-	307 (20%)	32.3	1	<0.0001	1.15
No	74	46	109	-	32	129	-	390 (92%)	225	16	251	-	136	617	-	1245 (80%)				
Diaphoresis																				
Yes	65	41	100	38	32	106	-	86 (18%)	487	14	213	136	124	341	-	753 (43%)	92.6	1	<0.0001	1.42
No	13	7	16	6	2	42	-	382 (82%)	72	18	83	80	50	450	-	1015 (57%)				
(Long) prodrome†																				
Yes	46	9	23	21	4	79	-	182 (39%)	172	21	96	160	57	611	-	1117 (63%)	89.7	1	<0.0001	1.66
No	32	39	93	23	30	69	-	286 (61%)	87	11	200	56	117	180	-	651 (37%)				
Blurred vision																				
Yes	26	6	30	8	1	26	-	97 (21%)	53	12	31	68	45	308	-	517 (29%)	13.5	1	0.0002	1.12
No	52	42	86	36	33	122	-	371 (79%)	206	20	265	148	129	483	-	1251 (71%)				
Palpitations																				
Yes	11	1	13	4	1	9	-	39 (8%)	26	8	38	2	7	119	-	200 (11%)	3.4	1	0.06	1.03
No	67	47	103	40	33	139	-	429 (92%)	233	24	258	214	167	372	-	1568 (89%)				
Supine syncope																				
Yes	9	2	-	6	-	-	-	17 (10%)	6	0	-	6	-	-	-	12 (2%)	18.1	1	<0.0001	4.23
No	69	46	-	38	-	-	-	153 (90%)	253	32	-	210	-	-	-	495 (98%)				
Syncope during effort																				
Yes	10	-	-	6	-	-	-	16 (13%)	7	-	-	2	-	-	-	9 (2%)	30.5	1	<0.0001	6.92
No	68	-	-	38	-	-	-	106 (87%)	252	-	-	214	-	-	-	466 (98%)				

* Number of patients per age category was calculated from the reported mean and SD of patients’ age

† Presence or prolonged presence of prodromal symptoms, or awareness of being about to faint

### Test populations

There were 4 test datasets.

*Canada and United Kingdom: Syncope Symptom Study,* ‘Calgary data’ [7,8,20]. Between January 1995 and July 2001, 670 patients (age 51±21 years) with at least one loss of consciousness were recruited from neurology, cardiology, pacemaker, arrhythmia and syncope clinics. All patients completed a 118-item questionnaire based on Calkins et al. [15], assessing symptom burden, provocative situations, peri-syncopal symptoms, symptoms thought to be associated with epileptic seizures, signs observed by bystanders and relevant medical history. Definitions of clinical diagnoses were tightly and prospectively defined; patients underwent electrophysiologic studies and where necessary also tilt table tests (excepting those with a clinically declared cause of syncope such as sustained VT during syncope) [20]. In total, 138 (21%) patients had cardiac syncope.

*Netherlands: Fainting Assessment Study* (*FAST*)*,* ‘Amsterdam data’ [21]. Between February 2000 and May 2002, 503 patients (age 52±19 years) presenting with a transient loss of consciousness at the neurology, cardiology, and internal medicine emergency department, or the cardiac emergency department of the Academic Medical Center in Amsterdam were enrolled. A standardised medical history was taken, using a questionnaire based on the European Society of Cardiology guidelines [1]. Clinical diagnoses were based on expert opinion after 2 years of follow-up, and documented arrhythmias where available. A total of 44 (9%) patients had cardiac syncope.

*Italy: Short-Term Prognosis of Syncope (STePS*)* Study,* ‘Milan data’ [9]. Between January and July 2004, 689 patients (age 60±21 years) presenting with syncope at emergency departments in one of four general hospitals in the Milan area were enrolled [9]. Cardiac syncope was mainly defined as a change in rhythm therapy or a documentation of a potential substrate for syncope. Only 37 (5%) patients had cardiac syncope.

*United States,* ‘Rochester data’ [2]. Between January 1996 and December 1998, 3877 patients (age 57±23 years) were evaluated for syncope at the Mayo Clinic in Rochester, Minn. Patients were referred from outpatient clinics, inpatient services, hospital emergency departments and other institutions. Clinical diagnoses were based on expert opinion, and documented arrhythmias where available. Cardiac syncope was defined as a documented symptom-rhythm correlation, or a potential substrate for syncope. In total, 424 (11%) patients had cardiac syncope.

### Distribution of predictors in test populations

The prevalence of predictors in each dataset, and how the predictors were distributed among patients with cardiac and non-cardiac symptoms, is shown in Table 3; not all predictors were surveyed in all centres. Structural heart disease was common among Calgary (21%) and Milan (25%) patients, but much less so among Amsterdam (10%) and Rochester patients (11%). Other pronounced differences between test datasets were in age and number of previous spells. Rochester and Milan patients were older than Calgary and Amsterdam patients. Having had only two spells or less was most common in Rochester patients. The median [interquartile range] for the number of previous spells was 4 [2-20] for Calgary patients, 3 [1-8] for Amsterdam patients and 1 [1,2] for Rochester patients. Having had a single faint occurred in 19% of Calgary patients, 27% of Amsterdam patients, 43% of Milan patients and 66% of Rochester patients. Having had at least 10 occurred in 33% of Calgary patients, 24% of Amsterdam patients, and 5% of Rochester patients.

**Table 3:** Distribution of age, gender and symptoms in relation to final diagnosis, for each patient population.

	Calgary data [7,8,20]				Amsterdam data [21]					Milan data [9]				Rochester data [2]		
	Cardiac syncope N=138(21)	Non-cardiac syncope N=525(79)	Total		Cardiac syncope N=44(10)	Non-cardiac syncope N=419(90)		Total		Cardiac syncope N=37(5)	Non-cardiac syncope N=652(95)	Total		Cardiac syncope N=424(11)	Non-cardiac syncope N=3453(89)	Total
Age																
<40 years	7 (5)	233 (44)	240 (36)		2 (5)	143 (34)		145 (31)		0 (0)	161 (25)	161 (23)		39 (9)	967 (28)	1006 (26)
40 to 60 years	20 (15)	141 (27)	161 (24)		10 (23)	148 (35)		158 (34)		4 (11)	137 (21)	141 (21)		48 (11)	648 (19)	696 (18)
≥60 years	111 (80)	151 (29)	262 (40)		32 (73)	128 (31)		160 (35)		33 (89)	354 (54)	387 (56)		337 (79)	1838 (53)	2175 (56)
Gender																
Male	106 (77)	246 (47)	352 (53)		35 (80)	226 (54)		261 (56)		22 (71)	272 (43)	294 (44)		256 (60)	1648 (48)	1904 (49)
Female	32 (23)	279 (53)	311 (47)		9 (20)	193 (46)		202 (44)		9 (29)	367 (57)	376 (56)		168 (40)	1805 (52)	1973 (51)
Struc heart disease																
Yes	81 (62)	62 (12)	143 (21)		18 (41)	29 (7)		47 (10)		16 (43)	158 (24)	174 (25)		135 (32)	279 (8)	414 (11)
No	49 (38)	463 (88)	512 (78)		26 (59)	390 (93)		416 (90)		21 (57)	494 (76)	515 (75)		289 (68)	3174 (92)	3463 (89)
Number of spells†																
Two spells or less	91 (66)	125 (24)	216 (33)		26 (62)	151 (41)		177 (43)		23 (62) †	370 (57) †	296 (43) †		262 (62)	2283 (66)	2545 (66)
More than two spells	47 (34)	400 (76)	447 (67)		16 (38)	218 (59)		234 (57)		14 (38)	282 (43)	393 (57)		162 (38)	1170 (34)	1332 (34)
Nausea																
Yes	30 (22)	229 (44)	259 (39)		6 (14)	129 (31)		135 (29)		−	−	−		64 (15)	808 (23)	872 (22)
No	108 (78)	296 (56)	404 (61)		37 (86)	287 (69)		324 (71)		−	−	−		360 (85)	2645 (77)	3005 (78)
Diaphoresis																
Yes	52 (38)	266 (51)	318 (48)		12 (29)	200 (48)		212 (46)		−	−	−		91 (21)	814 (24)	905 (23)
No	86 (62)	259 (49)	345 (52)		29 (71)	216 (52)		245 (54)		−	−	−		333 (79)	2639 (76)	2972 (77)
(Long) prodrome																
Yes	36 (26)	271 (52)	307 (46)		9 (20)	148 (35)		157 (34)		19 (51)	475 (73)	494 (72)		340 (87)	2976 (93)	3316 (92)
No	101 (74)	253 (48)	354 (54)		35 (80)	271 (65)		306 (66)		18 (49)	177 (27)	195 (28)		52 (13)	230 (7)	282 (8)
Blurred vision*																
Yes	33 (24)	215 (41)	248 (37)		−	−		−		−	−	−		6 (1)*	92 (3)*	98 (3)*
No	105 (76)	310 (59)	415 (63)		−	−		−		−	−	−		418 (99)	3361 (97)	3779 (97)
Supine syncope																
Yes	−	−	−		−	−		−		14 (38)	149 (23)	163 (24)		47 (11)	341 (10)	388 (10)
No	−	−	−		−	−		−		23 (62)	503 (77)	526 (76)		377 (89)	3112 (90)	3489 (90)
Syncope during effort																
Yes	−	−	−		10 (23)	42 (10)		52 (12)		0(0)	15 (2)	15 (2)		69 (16)	363 (11)	432 (11)
No	−	−	−		33 (77)	365 (90)		398 (88)		37 (100)	637 (98)	674 (98)		355 (84)	3090 (89)	3445 (89)

Results are given as number (

%) There were statistically significant differences in the overall prevalence of predictors, between the 4 centres (chi-square tests, p <0.001 for all symptoms/demographics). * ‘Blurred vision’ was not explicitly asked in the Rochester study. The term ‘blurred vision’ was extracted from a text field for patients’ comments. † In the Milan data, the number of spells was categorised as ‘One spell’ vs. ‘More than one spell’. Because this was the only information available, it was used as a proxy for ‘Two spells or less’ vs ‘More than two spells’.

### Performance of conditional probabilities model in test populations

Three analyses of the test populations were performed: 1) all 4 test populations; 2) a heavily resampled population to mitigate data collection biases; and 3) a parsimonious model.

**Figure 3:**
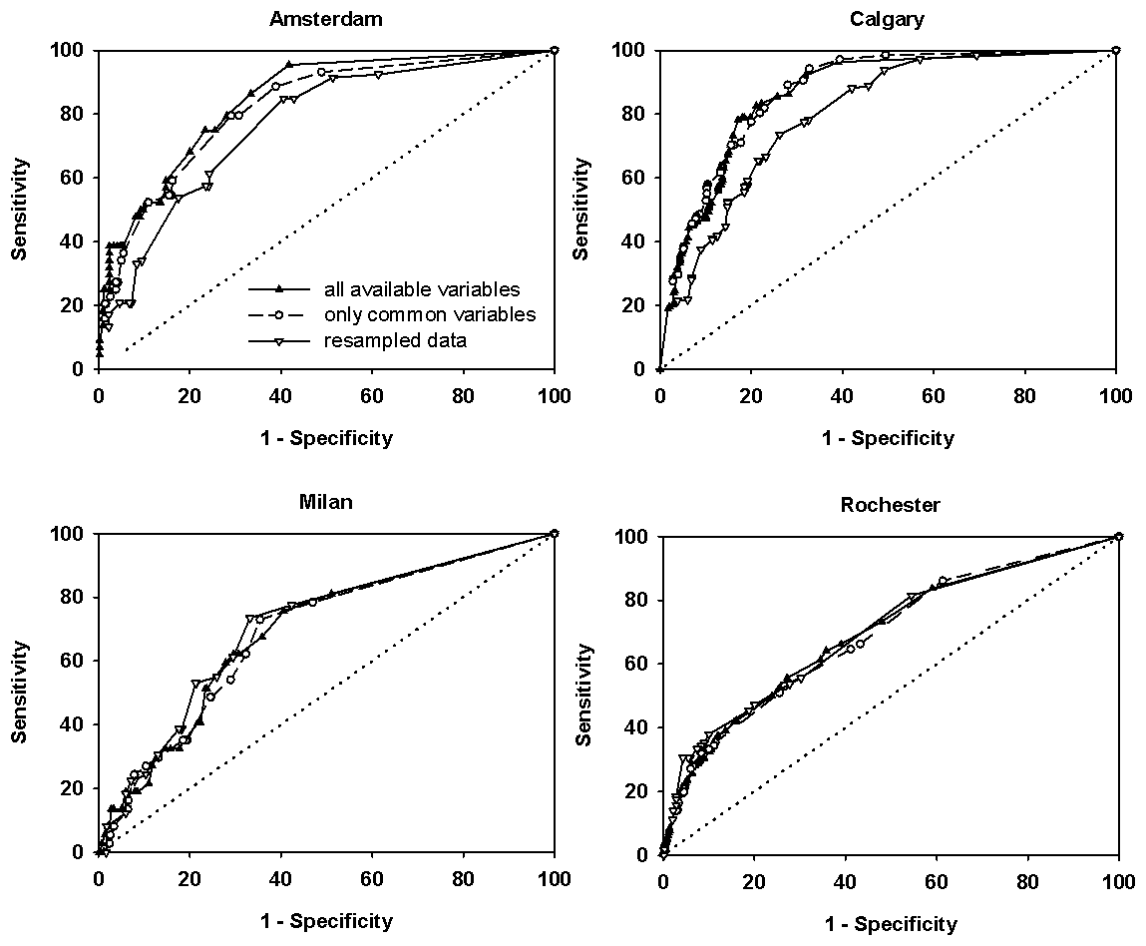
Receiver-operator curves (ROC) of the predicted probabilities against the actual outcome, for each dataset. Model results are shown using all available variables (closed triangles, solid line), for a parsimonious model using only variables common to all datasets (age, gender, structural heart disease, number of spells, and prodromal symptoms; open circles, short dashed line), and for a model using data resampled to create standard distributions of number of spells and age (categorical; open inverted triangles).

The conditional probabilities model, using all available information for each dataset, resulted in c-statistics of 0.87 for the Calgary data [7,8,20], 0.84 for the Amsterdam data [21], 0.72 for the Milan data [9] and 0.71 for the Rochester data [2] (Figure 3). With a cut-off value of the probability of cardiac syncope of 0.02 (selected to favour sensitivity over specificity), the sensitivity and specificity of the full model were 92% and 68% for Calgary, 86% and 67% for Amsterdam, 76% and 59% for Milan, and 73% and 52% for Rochester. Using only variables common to all datasets (age, gender, structural heart disease, number of spells, and prodromal symptoms) a more parsimonious model resulted in very similar c-statistics of 0.88, 0.83, 0.72 and 0.69, respectively.

**Figure 4:**
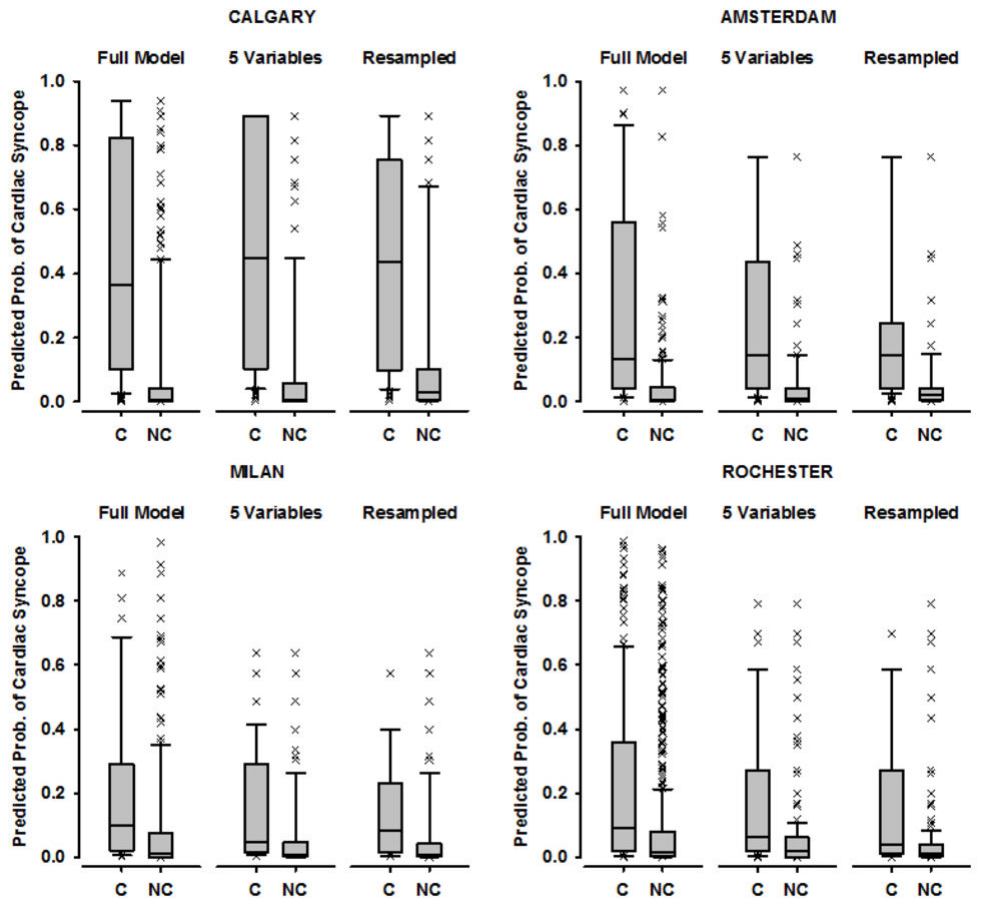
Predicted probability of cardiac syncope (median, inter-quartile range [box], 10-90^th^ percentile [whiskers] and outliers [crosses]) using three sampling methods. The full model includes all patients with a final diagnosis of cardiac syncope (C) and non-cardiac syncope (NC) using the full model; the parsimonious model uses only the 5 variables common to all datasets; and the resampled model uses resampled data with standardized distributions of age and number of spells.

In an effort to identify the source of discrepancy in model accuracy for the 4 test datasets, we sought to reduce accrual bias by standardizing the distribution of age and number of spells. The conditional probabilities model on the resampled data resulted in c-statistics of 0.80 for Calgary, 0.78 for Amsterdam, 0.73 for Milan and 0.69 for Rochester (Figure 3). There were great differences among centres in the mean predicted probability of cardiac syncope among cardiac syncope patients (Figure 4). However, for all locations, the predicted probability of cardiac syncope was greater in cardiac syncope patients than in non-cardiac syncope patients (Mann-Whitney rank sum test; p<0.001 for all comparisons).

## Discussion

We aimed to develop an international, literature-based model to predict cardiac syncope. This simple model of historical and demographic criteria distinguishes patients with cardiac syncope from patients with other causes of syncope, with accuracy in the range of the Framingham [23] and TIMI [24] scores. The accuracy of the simple model approaches the pooled estimates [25] of sensitivity and specificity of the San Francisco Syncope Rule used in emergency rooms for a variety of medical outcomes. However, there were differences between research centres in the predictive power of individual historical criteria, and therefore also in the predictive power of the overall model.

The inclusion of only 5 predictor variables preserved the overall accuracy of the model in all test datasets for cardiac syncope. They were age over 40 years, male sex, structural heart disease, no more than 2 spells, and brief or no prodromal symptoms. The parsimonious model is simpler, and therefore generally preferable. However it will not perform as well in specific settings, such as when arrhythmias are suspected in young people [26]. The summary of predictive powers in Table 2 contains useful data for these particular settings.

This study is the first literature-based model to identify patients with cardiac syncope based on historical and demographic criteria. Predictive models are generally data-driven, and may not be effective in other populations. This model is based on the results of 7 studies, conducted in 4 countries, and is more likely to be robust. To confirm this, model testing was performed on independent primary datasets from 4 countries. A further strength was the use of a conditional probabilities model taking into account the prior probability of cardiac syncope. Prior probabilities may differ among locations (and did), and taking this into account makes the model more suitable for use in different settings.

The model is surprisingly robust, given the differences among the derivation and test centres. It serves to identify the major clinical features that increase the likelihood for cardiac syncope. The results highlight the importance of male sex, advancing age, structural heart disease, recent onset, and few prodromal symptoms in predicting cardiac syncope. The model accuracy and robustness make it suitable as a risk stratification tool, although it cannot be reduced to a simple scoring rule. Clinical use of the model would involve implementation of the conditional probability tables and model algorithms as a smart-phone or desktop application, and these variables might also serve as the basis for development of a future, widely based score.

Clinical and physiologic features: The predictor variables are simple, and have familiar clinical and physiologic features. For example, male sex predicts cardiac syncope, which resonates with the lower likelihood of males having vasovagal syncope [27,28]. Advancing age predicts cardiac syncope, and it also associates with age-dependent diseases such as sick sinus syndrome, aortic stenosis, and myocardial infarction. Factors associated with non-cardiac syncope such as age < 40 years, a long prodrome, nausea, diaphoresis, and blurred vision resonate strongly with the demographic and clinical features of vasovagal syncope. The long prodrome and blurred vision is likely due to the relatively gradual decline of blood pressure, and the preceding diaphoresis reflects the intense cutaneous sympathetic activity that occurs with vasovagal syncope.

The complexities of health service and epidemiology studies of syncope have left the field with several large opportunities, which have only recently begun to be addressed. The main issues are the uncommon occurrence of documenting syncope in the act, the large differential diagnosis with quite varying severities of outcomes, the heterogeneous substrates that predispose to it, and the range of outcomes. Compounding this is a lack of uniform criteria and definitions for diagnosis, substrates, degree of certainty about diagnosis, variable approaches to diagnostic investigations, and variable definitions of outcomes. There are assumptions about the link between investigation results and true diagnosis, and therefore ongoing uncertainty that demonstrating electrical or structural substrates actually establish a diagnosis. As well, the relative uncommonness of any single diagnosis of any single form of cardiac syncope often leads to them being pooled together. With these problems it is not surprising that up to half of syncope goes undiagnosed in other than specialty centres. Finally, rare causes of syncope such as genetic arrhythmias do not figure prominently in most studies because of their rarity.

There have been three approaches to these problems. The first is to use very rigorous diagnostic criteria for each cause of syncope, exemplified by the Calgary Syncope Symptom studies [7,8,20] and the ISSUE ILR studies [29-32]. The second is to develop uniform criteria for outcomes, as is now the case for studies of syncope in the emergency department [33]. This study takes a third approach, more common and pragmatic, more probabilistic and integrative. It is based on 11 studies, and integrates the assumptions widely made in the practice of syncope. For example, it implicitly assumes that all cardiac syncope lacks the autonomic symptoms associated with vasovagal syncope, and that most vasovagal syncope has these symptoms. It implicitly assumes, as do its integrated studies, that identification of a substrate diagnoses the cause of syncope, and it simply accepts the varied definitions of cardiac syncope. It also implicitly accepts that not all syncope can be diagnosed. Although this approach lacks the rigor of some earlier studies, it has the strength of reflecting current styles and standards of practice. It also establishes a foundation for future, more tightly controlled studies.

### Limitations

There are several potential limitations. The model was based only on the data reported in a sufficient number of 7 studies [5,6,15-19], raising the possibility of inclusion bias due to the biases built into the care patterns of the involved health care systems. For example, a completely publicly funded system with easy access to health care facilities might increase the a priori probability of non-cardiac syncope. Second, not all test datasets included all model variables. Third, although interactions between variables in relation to the outcome were tested in logistic regression models (data not shown), dependence between predictors was not taken into account.

There are differences in terminology among centres in both derivation and test populations. Of the two studies used to derive conditional probabilities for structural heart disease, the term ‘heart disease’ was defined by one [19]; but not the other [16], and the definition “structural heart disease” varied among the reports. This report suggests the importance of international agreement on definitions, and for an international approach [25,33] to the development of decision rules that work well across all reasonably relevant clinical populations.

Fifth, diagnostic scores are only as good as the clarity and certainty of the reference diagnoses. The Calgary Syncope Symptom Study [7,8,20] used very precise a priori definitions, essentially requiring either a hemodynamically documented syncopal spell or an induced arrhythmia or faint. Others included inferential causes, such as sinus bradycardia documented at another time [2]. Differences in the certainty of the diagnoses might explain some of the differences among the model behaviours in the test populations.

Finally, there were differences among test populations in important variables such as age and number of spells [2,7-9,20,21]. However, resampling to create standardized distributions of number of age and spells did not equalize the model accuracy for the 4 test datasets. Whether other uncollected factors affect the association between predictors and diagnosis of cardiac syncope remains unknown.

### Conclusion

A literature-based conditional probability model using information on age, gender, structural heart disease, number of spells, (long) prodrome, nausea, diaphoresis, blurred vision, supine syncope and effort syncope identifies patients with cardiac syncope with moderate accuracy. Future studies might benefit from providing uniform definitions of predictors and outcomes, and collecting a wide range of patient characteristics to take into account inter-centre patient population differences.
